# Blind docking methods have been inappropriately used in most network pharmacology analysis

**DOI:** 10.3389/fphar.2025.1566772

**Published:** 2025-03-24

**Authors:** Xinhao Che, Lei Zhang

**Affiliations:** Frontier Science Center for Smart Materials Oriented Chemical Engineering, State Key Laboratory of Fine Chemicals, School of Chemical Engineering, Dalian University of Technology, Dalian, China

**Keywords:** network analysis, traditional Chinese medicine, blind docking, *in silico*, protein target

## Abstract

Network pharmacology methods have bridged the gap between traditional Chinese medicine (TCM) theory and contemporary pharmacological research and have been widely used in the study of multi-component, multi-target mechanisms of action of TCM. Molecular docking *in silico* is typically used after network analysis to validate the binding between protein targets and active components of TCM. However, unreasonable docking methods, especially the abuse of blind docking, have raised doubts about the docking results. This paper expresses concern about the above phenomenon based on a comprehensive assessment of the accuracy of the blind docking methods and calls for the correct use of docking methods to make the results of network analysis and experiments more convincing.

## 1 Introduction

Traditional Chinese medicine (TCM), especially herbs, has a history of thousands of years. These herbs are usually mixtures that act on multiple disease pathways and protein targets, containing various chemical components. TCM has shown its excellent efficacy in treating complex diseases, such as diabetes, cardiovascular diseases, chronic diseases, and the recently emerged COVID-19. However, because of their complex action mechanisms, it is difficult to determine which components play what therapeutic roles in the treatment process of a disease. The emergence of network pharmacology methods has greatly shifted the paradigm of pharmacological research on TCM (Zhai et al., 2025). The core step, biological network analysis, aims to uncover the synergistic and complex interactions between drugs, diseases, and therapeutic targets, which can reveal a modular relationship linking TCM phenotypes, molecular mechanisms, and herbal treatments. After that, molecular docking methods *in silico* are often used to validate the binding between targets and active components of TCM. It can be said that molecular docking results are strong support for the findings of network analysis and at the same time, serve as a preliminary consideration for whether further experimental validation is needed. However, blind docking methods have been inappropriately used to evaluate the interactions between targets and active components, especially in network analysis research, which often leads to false positive docking results. Blind docking means using a sufficiently large search space to cover the entire protein structure to search for possible binding conformations of small molecule ligands when the actual binding site is unknown. Although this method may be effective sometimes (Hetényi and Van Der Spoel, 2006), it is still inappropriate to directly evaluate the interactions between molecules using the blind docking results. Due to the diversity and complexity of protein structures, small molecule ligands are likely not to be docked to the actual active sites, but rather docked to other regions with low binding energy because almost all docking algorithms are based on the principle of energy minimization between the ligand and the receptor. Therefore, the docking results are also unreliable.

Combined with a brief review of the docking methods in network pharmacology research published in *Frontiers in Pharmacology* and a comprehensive assessment of the accuracy of blind docking methods, we express our concerns about the abuse of blind docking methods. It is our sincere hope that more rigorous docking procedures can be used to make the network analysis results more scientific.

## 2 Brief review of the docking methods in network pharmacology

We reviewed all the 35 research papers related to docking methods that contain “network pharmacology” in the title. They were published last year (2024) in *Frontiers in Pharmacology*. All statistical results are summarized in Table 1. Of course, there is also an abuse of blind docking methods in network pharmacology studies published in other journals and similar analysis results and conclusions can also be obtained.

**TABLE 1:** Molecular docking methods used in 35 network pharmacology research papers.

Index	DOI	Docking software	blind docking	Docking box size (Å)	Affinity threshold (kcal/mol)
1	10.3389/fphar.2024.1261772	Autodock	unknown	unknown	−4.25
2	10.3389/fphar.2024.1282361	Autodock Vina	unknown	unknown	−5.0
3	10.3389/fphar.2024.1290398	Autodock Vina	No	unknown	−5.0
4	10.3389/fphar.2024.1310009	iGEMDOCK	suspicious	unknown	—
5	10.3389/fphar.2024.1328334	Autodock Vina	unknown	unknown	−5.0
6	10.3389/fphar.2024.1345415	PyRx	suspicious	unknown	−5.0
7	10.3389/fphar.2024.1352907	Glide	No	unknown	—
8	10.3389/fphar.2024.1355644	Autodock Vina	No	unknown	−5.0
9	10.3389/fphar.2024.1359427	Autodock Vina	suspicious	unknown	−5.0
10	10.3389/fphar.2024.1361379	Autodock	suspicious	unknown	−1.2
11	10.3389/fphar.2024.1363415	Autodock	unknown	unknown	−5.0
12	10.3389/fphar.2024.1366279	Autodock	Yes	unknown	—
13	10.3389/fphar.2024.1388540	Autodock Vina	suspicious	unknown	−4.2
14	10.3389/fphar.2024.1395014	Autodock Vina	No	18 × 22 × 26	—
15	10.3389/fphar.2024.1395160	Autodock Vina	No	unknown	−1.2
16	10.3389/fphar.2024.1403864	Autodock Vina	unknown	unknown	−5.0
17	10.3389/fphar.2024.1405596	Autodock Vina	unknown	unknown	−5.0
18	10.3389/fphar.2024.1407525	Autodock Vina	Yes	60 × 60 × 60	−6.0
19	10.3389/fphar.2024.1414856	Autodock Vina	suspicious	unknown	−7.0
20	10.3389/fphar.2024.1415422	CB-DOCK	suspicious	unknown	−7.0
21	10.3389/fphar.2024.1431391	Autodock	unknown	unknown	—
22	10.3389/fphar.2024.1446707	Autodock	suspicious	unknown	0.0
23	10.3389/fphar.2024.1448308	Autodock Vina	Yes	60 × 42 × 51	—
24	10.3389/fphar.2024.1448381	Autodock Vina	unknown	unknown	−5.0
25	10.3389/fphar.2024.1451300	Autodock Vina	suspicious	unknown	—
26	10.3389/fphar.2024.1457012	Autodock Vina	Yes	42 × 41 × 45	—
27	10.3389/fphar.2024.1466114	Autodock Vina	unknown	unknown	−3.0
28	10.3389/fphar.2024.1480562	Autodock	suspicious	unknown	−2.0
29	10.3389/fphar.2024.1481091	unknown	suspicious	unknown	−5.0
30	10.3389/fphar.2024.1485915	unknown	unknown	unknown	−5.0
31	10.3389/fphar.2024.1486563	Autodock Vina	unknown	unknown	−5.0
32	10.3389/fphar.2024.1487474	Autodock Vina	suspicious	unknown	—
33	10.3389/fphar.2024.1495343	Autodock Vina	suspicious	unknown	−5.0
34	10.3389/fphar.2024.1496664	Autodock	No	30 × 30 × 30	−5.0
35	10.3389/fphar.2024.1517386	Glide	unknown	unknown	—

As shown in Table 1, Autodock Vina (Trott and Olson, 2010) and Autodock (Morris et al., 2009) is still the most widely used docking software. Their open-source and user-friendly features are favored by researchers. However, whether blind docking was used is not mentioned in many papers, nor are detailed docking parameters provided, such as the size and the center position of the docking box, which makes it difficult to reproduce the docking results and raises doubts about the reliability. Only 6 papers docked ligands to a specific binding site. Among these papers, only 2 papers used binding site detection software/algorithms before the docking step. 3 papers considered the location of the original crystal ligand in proteins as a binding site. The last paper did not disclose further details of binding site settings. Through careful inspect of the docking methods and results, in addition to the 4 papers that mentioned the use of blind docking methods, there are also 13 papers that are suspicious of using blind docking methods because the protein-ligand interaction diagrams in these papers show that different small molecule ligands, usually active monomers in TCM, were docked to different domains of a protein target, rather than the same active site, which is quite rare unless a protein target is multifunctional and binds to different ligands to exert different physiological functions. Furthermore, the “affinity threshold” in the table means that if an absolute value of the binding affinity (docking score) is above this threshold, the ligand binds well with the protein. Otherwise, the interactions are weak, as claimed in the above papers. But paradoxically, there is no unified standard for this threshold. It seems that −5.0 kcal/mol is accepted in most papers. In fact, there is no standard to distinguish between “good” and “bad” binding. A positive control molecule is needed to demonstrate that the docking ligand has a better binding to the protein target but unfortunately, we did not find positive controls used in any papers. It should also be noted that although the affinity provided by most docking software is in kcal/mol, the calculation results from different software are not comparable. Therefore, applying an affinity threshold to the results from different docking software is quite absurd.

## 3 Comprehensive assessment of the accuracy of blind docking methods

**FIGURE 1:**
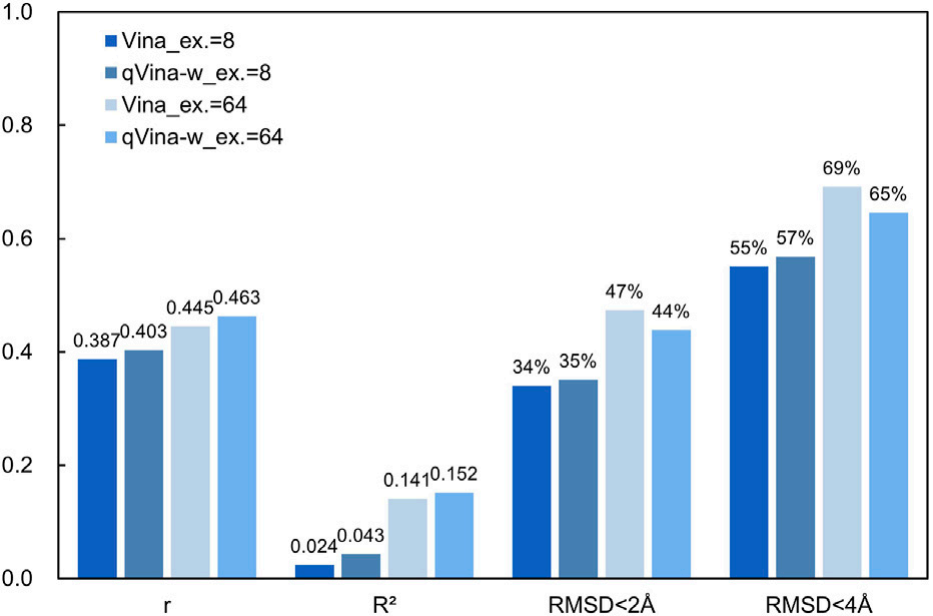
Accuracy of blind docking on the CASF-2016 dataset.

In order to fully illustrate that blind docking is significantly less accurate than docking with specific binding sites, the accuracy of blind docking using two popular docking software, Autodock Vina 1.2.5 (Vina) and QuickVina-w (qVina-w) (Hassan et al., 2017), was tested on the CASF-2016 dataset (Su et al., 2019), and the results are summarized in Figure 1. QuickVina-w was reported to be more suitable for blind docking, thanks to its more thorough search of the protein structure. The CASF-2016 dataset contains 285 pairs of high-quality protein receptors and their ligands, which is a common benchmark for docking performance tests, but the blind docking performance has not been tested. The “exhaustiveness” (*ex.*) parameter in the variants of Vina software will also affect the docking results, which represents how many conformations are sampled for the input ligand (Che et al., 2023). The default value of 8 and a much higher value of 64 were selected for this test. In each docking, the center of the docking box is set as the geometric center of the protein and the size of the box is slightly larger (2 Å) than the length, width, and height of each protein, in order to provide more rotatable space for ligands that may bind to the protein surface. All the proteins were prepared using AutodockTools 1.5.6 (Morris et al., 2009), which contains retaining a single peptide chain for homomeric oligomers (unless the ligand binds between two homologous chains), removing all crystal water molecules and solvent molecules, adding all hydrogen atoms to the protein, calculating Gasteiger charges of each atom, converting the format of the original files to *pdbqt* that can be recognized by Vina, and so on. All the small molecule ligands were prepared using OpenBabel 2.4.1 (O’Boyle et al., 2011), which contains correcting the molecular protonation states under the pH = 7.4 (the pH in human body fluid environment is usually 7.35∼7.45) and converting the format of the ligand files to *pdbqt*.

As shown in Figure 1, the correlation between the calculated binding affinity and the experimental values is very low. The reported Pearson Correlation Coefficient (r) of Vina on the CASF-2016 dataset is 0.604 (0.539 ∼ 0.659, 90% confidence interval) when docking with specific binding sites (Su et al., 2019), while that is 0.387 when using blind docking methods. The extremely low Coefficient of Determination (R^2^) indicates that the affinity obtained from blind docking hardly reflects the actual ligand-receptor binding strength, while in our tests, using the docking methods with specific binding sites can achieve an R^2^ of about 0.5. A higher exhaustiveness parameter slightly improves accuracy, but results in an 8-fold increase in computational time. QuickVina-w also slightly improves the accuracy of blind docking, but it is limited.

What’s more, blind docking results in many ligand molecules not binding at their actual binding sites, which is reflected in the large Root Mean Squared Deviation (RMSD) values between the docking conformations and the crystal conformations. In our tests, the median values of RMSD on the CASF-2016 dataset are 3.370Å (Vina, *ex.* = 8), 3.119Å (qVina-w, *ex.* = 8), 2.207Å (Vina, *ex.* = 64), and 2.476Å (qVina-w, *ex.* = 64), respectively. They are all below the strict standard of 2 Å (Buttenschoen et al., 2024). We calculated the ratio of docking conformations with RMSD less than 2 Å and less than 4 Å among all the docking results. As can be seen in Figure 1, this ratio is between 34% ∼ 47% with the RMSD threshold of 2 Å, while that is between 55% ∼ 69% with the RMSD threshold of 4 Å. It is reported that the success rate (RMSD <2 Å) of docking with specific binding sites is 90.2% when docking with specific binding sites using Vina on the same dataset (Su et al., 2019). As the docking region covers the whole protein structure, the sampling space of ligand conformations is huge, which is the fundamental reason for the serious decline of the accuracy of blind docking. The results of blind docking and the ligand-receptor interaction diagrams are unreliable in many cases. Although many efforts have been made to develop more accurate and more thorough conformation search algorithms, blind docking results are reliable only for those structurally simple proteins at present.

## 4 Discussion

As network analysis methods become more and more developed, it has contributed significantly to the pharmacological research of TCM. Molecular docking builds a bridge between network analysis and experimental validation. Although there is still a gap between the current molecular simulation methods and experimental results, more accurate computational results remain a goal that researchers have been striving for. More scientific docking algorithms, more advanced docking software, and even artificial intelligence-assist docking prediction are continuously narrowing this gap. However, inappropriate docking procedures and parameters will make the results completely off the actual mark, which is unrelated to the docking software itself. A scientific docking result can reduce unnecessary experimental trials, saving manpower, resources, and financial costs, while an unreliable docking result may undermine the network analysis, leading to inconsistency between the network analysis and experiments. Specifically, blind docking often leads to false-positive results, which means that the active ingredient in TCM shows a high affinity score with the protein target, while in reality, it cannot bind to the target stably. We are concerned about the blind docking methods currently used in most network pharmacological research and call for the correct use of docking methods to make the results more convincing. Admittedly, the inappropriate use of docking methods also exists in other research fields, but it is so common in network pharmacology research. Some specific docking suggestions are as follows.

(1) We strongly recommend reading the paper by Yu-Chian Chen (Chen, 2015). Chen is one of the earliest scholars focusing on the application of network pharmacology in TCM. In the paper “Beware of docking”, numerous potential issues and considerations that may arise during a docking process are summarized, which can greatly help relevant scholars achieve a “perfect” docking.
(2) Try to avoid using blind docking methods and more detailed parameter settings and steps during a docking process should be mentioned in the paper. For the genes/targets that frequently appear in metabolic pathways, especially those with experimentally determined crystal structures, a comprehensive literature review is necessary to identify the specific functional site on the protein.
(3) If there is indeed no binding site information available for a specific target, a pocket detection software/algorithm is needed, such as the SiteMap module in the Schrödinger software (Schrödinger Release 2024–4: SiteMap; Schrödinger, LLC: New York, NY, 2024) or something similar. And then, docking should be performed carefully to ensure that an active molecule binds to the actual site as expected. A double validation of docking results is also encouraged, which means using a second software to confirm binding sites identified with one software. More detailed docking parameter settings can be referenced from the paper (Che et al., 2023). Or, we also encourage the use of docking methods specifically designed for blind docking, especially the popular AI-based methods such as AlphaFold 3 and RoseTTAFold All-Atom, which offer significantly improved accuracy compared to traditional docking methods.
(4) Be cautious about the affinity results given by the docking software. Multiple validations of the results can be performed using different docking software/algorithms, and it is better to further verify the ligand-receptor dynamic binding stability through subsequent molecular dynamics simulations and binding free energy calculations. For experimental journals, experimental verification of the docking results is necessary, which is the most convincing way. A proposed pharmacological action mechanism is required.
(5) In the longer term, we plan to develop a network pharmacology database at the binding site level, forming a comprehensive network structure of compound-binding site-target-pathway-disease, so as to contribute to the further development of network pharmacology.

## 5 Conclusion

Blind docking methods have been inappropriately used in most network pharmacology analysis, which may undermine the scientific validity of network analysis. In this commentary, we briefly reviewed 35 papers involving network analysis and docking validation and pointed out some potential risks in the blind docking methods used in the above papers. After a comprehensive evaluation of the accuracy of blind docking, we provided detailed improvement suggestions. Hope that network pharmacology can play a more important role in TCM.

## Data Availability

The dataset CASF-2016 used for the evaluation of the performance of the blind docking methods in this paper can be found in http://www.pdbbind.org.cn/casf.php/.
